# Venous endothelial injury in central nervous system diseases

**DOI:** 10.1186/1741-7015-11-219

**Published:** 2013-10-11

**Authors:** Jonathan S Alexander, Leonard Prouty, Ikuo Tsunoda, Chaitanya Vijay Ganta, Alireza Minagar

**Affiliations:** 1 Department of Molecular and Cellular Physiology, LSU Health Sciences Center, 1501 Kings Highway, Shreveport, LA 71130-3932, USA; 2 Department of Pathology, LSU Health Sciences Center, 1501 Kings Highway, Shreveport, LA 71130-3932, USA; 3 Department of Microbiology and Immunology, LSU Health Sciences Center, 1501 Kings Highway, Shreveport, LA 71130-3932, USA; 4 Department of Neurology, LSU Health Sciences Center, 1501 Kings Highway, Shreveport, LA 71130-3932, USA

**Keywords:** Venous, MS, CNS, ADEM, Inflammation

## Abstract

The role of the venous system in the pathogenesis of inflammatory neurological/neurodegenerative diseases remains largely unknown and underinvestigated. Aside from cerebral venous infarcts, thromboembolic events, and cerebrovascular bleeding, several inflammatory central nervous system (CNS) diseases, such as multiple sclerosis (MS), acute disseminated encephalomyelitis (ADEM), and optic neuritis, appear to be associated with venous vascular dysfunction, and the neuropathologic hallmark of these diseases is a perivenous, rather than arterial, lesion. Such findings raise fundamental questions about the nature of these diseases, such as the reasons why their pathognomonic lesions do not develop around the arteries and what exactly are the roles of cerebral venous inflammation in their pathogenesis. Apart from this inflammatory-based view, a new hypothesis with more focus on the hemodynamic features of the cerebral and extracerebral venous system suggests that MS pathophysiology might be associated with the venous system that drains the CNS. Such a hypothesis, if proven correct, opens new therapeutic windows in MS and other neuroinflammatory diseases. Here, we present a comprehensive review of the pathophysiology of MS, ADEM, pseudotumor cerebri, and optic neuritis, with an emphasis on the roles of venous vascular system programming and dysfunction in their pathogenesis. We consider the fundamental differences between arterial and venous endothelium, their dissimilar responses to inflammation, and the potential theoretical contributions of venous insufficiency in the pathogenesis of neurovascular diseases.

## Introduction

The human central nervous system (CNS) can be affected by a number of inflammatory demyelinating diseases. This covers a wide range of clinically and neuropathologically heterogeneous conditions, which share some clinical characteristics, but possess distinguishing immunopathological features. Multiple sclerosis (MS) and acute disseminated encephalomyelitis (ADEM) are two of the most prominent of these inflammatory diseases. Although these conditions have different root causes, mechanisms, and courses, their underlying neuropathologies both exhibit perivenular demyelination. This strikingly significant important finding points to significant contributions by veins in these conditions, and suggests that venous dysfunction or vein-targeted disease processes, (rather than arterial pathology or injury) contributes to the development of these inflammatory CNS diseases. Unlike the cerebral arterial system, the spatial organization of cerebral venous networks is more complex and more often asymmetric, with greater structural heterogeneity than cerebral arterial anatomy. Consequently, this half of the circulatory system has been far less studied and understood [1].

Several reviews [2] have evaluated clinical and structural factors in venous contributions to neurologic diseases. In addition to the inflammatory-based view of the pathogenesis of these demyelinating diseases, the past few years has witnessed the emergence of a controversial view about MS. Could neurological disease processes such as MS be triggered or intensified in part through venous vascular disturbances? Although venous disturbances in particular have long been recognized in several forms of neurological disease, we are only recently appreciating how venous structure, programming, and responses contribute to specific features of these diseases. The concept that neurologic disease can be influenced by structural or functional abnormalities of the CNS venous system has raised intense worldwide debate among researchers, with many investigators arguing against its existence. Controlled, careful clinical studies are needed to validate when and how vascular alterations can contribute to forms of CNS injury and inflammation. Here, we provide a discussion on the potential pathogenesis of these diseases, with emphasis on venous endothelial dysfunction in MS, ADEM, and other forms of neuroinflammation.

### Pathophysiology of MS with emphasis on venous dysfunction

MS is a group of immune-mediated demyelinating syndromes associated with neurodegeneration in the human CNS, which causes significant neurological disability in largely younger adults (Noseworthy [3], Compston and Coles [4]). MS can affect both gray and white matter in any region of the CNS. Four distinct clinical patterns of MS are recognized: relapsing-remitting (RRMS), primary progressive MS (PPMS), secondary progressive MS, and progressive relapsing MS. To date, vascular studies in MS have investigated cerebrovascular capillary and large vessel venous endothelial cells that are not always derived from (or strictly relevant to) the CNS [5-7]. There has been less research into the arterial and venous differences in MS. Despite these limitations, vascular contributions in MS do appear to support the notion of the vasculature being an initiating target in MS etiology and not simply a bystander presentation of other disease processes. Perhaps the strongest support for this is the number of MS therapies that have been developed, which target leukocyte binding to activated endothelial cells, a central component of the blood-brain barrier (BBB). Vascular abnormalities in MS also include evidence of increased circulating markers of vascular inflammation, [8-10], which can lead to inflammatory challenges that initiate or exacerbate CNS injury. Magnetic resonance imaging (MRI) studies in MS also indicate longer mean blood-flow transit times, which indicates relatively lower cerebral blood flow in MS plaques, as well as decreased cerebral blood flow and prolonged mean transit time in normal-appearing white matter (NAWM). Decreases in brain blood flow increase with age in MS, with severity and form of MS (PPMS > RRMS) both of which may intensify ischemic injury [2,9,11]. Importantly, in apparently NAWM, the state of ischemia appears to occur before the appearance of plaques [10]. It is unclear whether diminished cerebral flow represents restricted perfusion (arterial-sided) or outflow restriction (venous influences). Further, venous blood exiting the cerebral veins of patients with MS in susceptibility-weighted imaging (also known as blood oxygen level dependent imaging) suggests lower net tissue oxygen consumption compared with controls [12], which points to disturbances in energy metabolism. These findings suggest an early role for vascular disturbances in MS, which may trigger later injury processes, but do not specifically indicate underlying vascular defects as their basis.

There are several significant differences between venous and arterial endothelial cells, which may play a role in increased susceptibility of the venous compartment as related to MS, ADEM, or chronic venous insufficiency (CVI); these differences include arteriovenous programming, flow shear-dependent gene expression, hemodynamic effects on autacoids and venous valve organization.

### Arterial versus venous differences in response to inflammation

The adhesive qualities of arterial and venous endothelial cells can be modified by inflammation or disease. In comparison with the arterial environment, lower venous shear stresses combined with increased venous endothelial permeability and responsiveness (to at least some inflammatory mediators) may make venules and veins more susceptible to developing inflammation. For example, Kalogeris et al., [13] showed that cytokine-responsive endothelial cell adhesion molecule (ECAM) responses toward cytokine exposure were higher in venous endothelium than in corresponding (umbilical) arterial endothelium, and also supported higher (venous) endothelial rates of binding of monocytes. Tumor necrosis factor (TNF)-α and lipopolysaccharide (LPS) were seen to significantly increase monocyte binding to venous, but not arterial endothelium *in vitro*. Furthermore, neither TNF-α nor LPS induced surface expression of vascular cell adhesion molecule (VCAM)-1 or E-selectin in arterial endothelium, and TNF did not induce VCAM-1 mRNA in arterial endothelium. Lastly, as a VLA-4 blocking antibody prevented about 75% of TNF-α-stimulated monocyte adhesion in venous endothelium, VCAM-1 dependent adhesion may be particularly important in TNF-α response. Interestingly, despite a TNF-α-mediated increase in surface-expressed intercellular adhesion molecule (ICAM)-1 in arterial endothelium, TNF-α did not increase monocyte adhesion to arterial endothelium. Amberger *et al*. [14] also found that venous endothelium (umbilical and saphenous veins) expressed higher levels of ICAM-1, VCAM-1, and E-selectin than arterial endothelium in response to TNF-α, interleukin (IL)-1β, and LPS, but lower levels of adhesion molecule responses to low density lipoprotein. Therefore, venous endothelium appears to be innately programmed for higher adhesive responses compared with arterial endothelium. Similarly, Wang and Feuerstein [15] showed that ischemia is a potent, albeit slower stimulus for ICAM-1 and E-selectin expression in the brain, potentially linking reduced blood flow in lesions and NAWM with immune cell infiltration.

With respect to underlying BBB differences between venous and arterial endothelium, we have previously reported that, compared with arterial endothelial cells, venous endothelial cells expressed more vascular endothelial (VE)-cadherin at the mRNA and protein levels Kevil et al., [16]. Conversely, arterial endothelial cells were found to express eighteen-fold more occludin at the protein and nine-fold more at the mRNA level. Occludin was also seen to be more organized at inter-endothelial junctions in umbilical arterial endothelial cells compared with umbilical venous endothelial cells Kevil et al., [16]. Interestingly, disturbances in flow direction, but not necessarily shear, modulate claudin-5, another component of tight junctions, which also shows arteriovenous endothelial heterogeneity, with arterial endothelium expressing higher levels of claudin-5 than venous endothelium [17]. Claudin-5 is also induced by estradiol [18], which could be a factor in gender-specific differences in BBB or disease incidence. Endothelial expression of junctional components (and barrier) also depends on other cells in the neurovascular complex, such as astrocytes [19], as well as the effects of inflammatory mediators on these support cells and the endothelium [20].

Although arterial and venous endothelial cells are heterogeneous, ‘arterialization’ of venous endothelial grafts (for example, in coronary artery bypass grafts), also suggests that fluid shear, oxygen tension differences, and other environmental factors can remodel transplanted veins into arterial homologs, and significant post-natal arteriovenous plasticity may be induced under different circumstances. Because each vascular type exhibits different relative responses to different types of injury and inflammatory stimuli, chronically altered shear stress or retrograde flow may lead to injury, which could reflect the mechanical trauma of the intima, as well as a shear-dependent remodeling of vessels exposed to shear forces. Adamson *et al*. recently showed that retrograde flow, rather than shear forces, diminishes the venous endothelial solute barrier by decreasing the organization of endothelial junctional VE-cadherin and occludin, a finding supporting the concept that abnormal flow patterns can dysregulate endothelial barrier properties Adamson et al., [21]. It is still unclear whether transvenular leukocyte extravasation is also enhanced by retrograde flow.

Zakkar *et al*. reported that induction of a pro-inflammatory phenotype on venous endothelial cells involves the phosphorylation-dependent activation of p38 mitogen-activated protein kinase (MAPK), which leads to the production of chemokines, including IL-8 and monocyte chemotactic protein-1 Zakkar et al., [22]. Thus, veins exposed to shear undergo activation of p38 MAPK, which may lead to inflammation. By contrast, shear exposure in arteries has been shown to lead to induction of MAPK phosphatase (MKP)-1, which decreases MAPK signaling. In that report, Sakkar et al. demonstrated that dexamethasone could induce expression of MKP-1 in venous endothelium, effectively recapitulating the protective effect of shear seen in arterial endothelium exposed to laminar shear stress. These events require extracellular regulated kinase (Erk)1 and Erk 2, cAMP response element binding, and oxidant signaling. The current use of dexamethasone in MS might therefore correct an abnormal flow-mediated activation of venous inflammatory programs and fully integrate components of the BBB.

### Is there altered hemodynamic signaling in venous inflammation?

In what other ways might flow disturbances lead to hyperactivation of inflammatory responses in the venous circulation? Krueppel-like factor (KLF)2 and KLF4 are shear-dependent transcription factors that suppress endothelial responses to inflammatory stimuli, such as TNF-α [23], and several important shear-sensing mechanisms rely on KLF2 and KLF4 to provide important links between laminar fluid shear and the maintenance of a quiescent endothelial phenotype (Table 1). Conversely, disturbances in normal flow patterns might increase inflammation through KLF2/4 dysregulation. Laminar shear regulates KLF2 by promoting the phosphorylation and nuclear export of histone deacetylase (HDAC)5, a process under the control of Ca^2+^/calmodulin. This process partitions HDAC5 from myocyte enhancer factor-2, which then triggers KLF2 expression. Wu *et al*., [24] also showed that laminar shear suppressed miRNA-92a, an endogenous inhibitor of KLF2 [24] and KLF4 [25]. KLF4 expression is also suppressed by HDACs, and is de-repressed by the HDAC inhibitor trichostatin-A (Table 1) [26]. KLF2 expression varies dramatically between arteries and veins, with arteries expressing about four-fold more KLF2 than their corresponding veins [23]. Liu *et al*. also found that freshly isolated arterial endothelium expressed higher levels of KLF2 than did venous endothelium, consistent with greater KLF2 arterial responses to fluid shear stress [23]. KLF2 was shown to be suppressed by inflammatory stimuli such as IL-1β [27], unlike KLF4, which paradoxically was activated by TNF-α, IL-1β and interferon (IFN)-γ [28], as well as shear. Venous cells exposed to shear also increase KLF2 expression (compared with static cultures) [29]. KLF2 is an important shear-activated transcription factor which upregulates endothelial nitric oxide synthase (eNOS) and thrombomodulin (TM) expression and reduces plasminogen activator inhibitor-1 (PAI-1) expression [30]. KLF2 also suppresses IL-1β induced endothelial VCAM-1 and E-selectin expression and TNF-α induction of tissue factor (TF) [27,30,31]. Shear-induced expression of KLF2 also suppresses activation of the pro-inflammatory transcription factors activator protein-1, nuclear factor κB Das et al., [32], and activating transcription factor 2 Fledderus et al., [33]. Importantly, induction of KLF2 in venous endothelium reduced TNF-α-induced E-selectin and VCAM-1. Shear-activated KLF2 also maintains endothelial quiescence by suppression of TNF-α receptors, upregulation of eNOS [27] and by decreasing angiopoietin-2 content in endothelial Weibel-Palade bodies [34]. KLF4 is similarly induced in endothelial cells by laminar shear stress and interestingly by inflammatory cytokines. Like KLF2, KLF4 also induces eNOS and TM, and suppresses endothelial VCAM-1 expression. KLF4 activation also decreases thrombus formation by downregulating tissue factor expression [28], and KLF4 also downregulates plasminogen activator inhibitor (PAI)-1. Therefore venous hemodynamic flow disturbances that lead to silencing of anti-inflammatory KLF2/KLF 4 programs might increase inflammation through altered endothelial barrier, leukocyte binding, and hemostasis [35]. Interestingly, 3-hydroxy-3-methylglutaryl-coenzyme A reductase statin drugs have recently been described as activators of KLF2 [36] and KLF4 [37], and may restore or maintain atheroprotective programs suppressed by abnormal venous flow fluid shear patterns. Statin activation of KLF2 also induces hemoxygenase-1, an important suppressor of inflammation [38]. Similarly, other drugs that maintain KLF2/4, such as HDAC inhibitors, might represent novel treatments for treating abnormal signaling in venous (and also arterial) endothelium produced by flow abnormalities.

**Table 1:** Pathophysiology of venous abnormalities in multiple sclerosis and potential therapeutic strategies

**Pathophysiology**	**Involved molecules**	**Potential intervention**	**Potential treatments**	**References**
Higher venous endothelial responses to inflammation	Cytokines, chemokines, adhesion molecules, occludin	Induction of MKP-1, protection against shear stress responses	Dexamethasone	[16,39]
Altered hemodynamic signaling in venous inflammation	KLF2, KLF4, eNOS, VCAM-1, PAI-I, TNF-α	Activation of KLF2 and KLF4	Statin drugs, HDAC inhibitors (for example, trichostatin-A)	[8,30,36,40]
BBB dysregulation	NMDA receptor, MMP-8, MMP-9, p38 MAPK	MMP inhibitor, p38 MAPK inhibitor	Doxycycline, minocycline, SB 239063	[41-44]
Venous remodeling	Collagens, iron, TGF-β1, p38 MAPK, VEGF, TIMP, MMP	p38 MAPK inhibitor, TGF modifier, angiotensin antagonist, anti-angiogenic drug, MMP inhibitor	Drugs (dilamapimod, avotermin, candesartan, bevacizumab, cavtratin, doxycycline, desferrioxamine)	[8,45-47]
Hemodynamic abnormality, CCSVI	PGI_2_, NO, EDHF	Venous pressure reduction	venoplasty	[48,49]

*Abbreviations:BBB* blood-brain barrier, *CCSVI* chronic cerebrospinal venous insufficiency, *EDHF* endothelium-derived hyperpolarizing factor, *eNOS* Endothelial nitric oxide synthase, *HDAC* histone deacetylase, *KLF* Krueppel-like factor, *MAPK* mitogen-activated protein kinase, *MKP* mitogen-activated protein kinase phosphatase, *MMP* matrix metalloproteinase, *MS* multiple sclerosis, *NMDA* N-methyl-D-aspartate, *NO* nitrous oxide, *PAI* plasminogen activator inhibitor, *PGI*_*2*_ prostaglandin I_2_ (prostacyclin), *TGF* transforming growth factor, *TIMP* tissue inhibitor of metalloproteinase, *TNF* tumor necrosis factor, *VCAM* vascular cell adhesion molecule, *VEGF* Vascular endothelial growth factor.

### Is the blood-brain barrier altered by factors induced in neurodegenerative disorders?

Several factors present in MS may dysregulate BBB in such a way that when presented with altered flow or pressure gradients, significant disturbances in BBB could be produced. It is now fairly well accepted that VE cells express N-methyl-D-aspartate (NMDA) and metabotropic receptor complexes, which contribute to regulation of the BBB. Glutamate is increased in the cerebrospinal fluid (CSF) in patients during relapse [50] consistent with its release during CNS injury. Binding of glutamate to endothelial NMDA receptor elevates intracellular oxidants [44] and disturbs the microvascular barrier [51], effects that may exacerbate matrix metalloproteinase (MMP)-9-mediated proteolysis of tight junctional components in the BBB, such as occludin Wachtel et al., [52] and claudin-5 [53]. Serum MMP-8 and MMP-9 are correlated with decreased numbers of T2-weighted lesions. [41]It is unclear what the sources of these MMPs are in this setting. Importantly, MMP-9 is known to proteolyze occludin, a tight junction target of the BBB Wachtel et al., [52]. Interestingly, it has been reported that, compared with laminar shear stress, oscillatory flow increases endothelial MMP-9 expression [54], and might alter the BBB in regions experiencing abnormal flow. In Alzheimer’s disease, β-amyloid appears to help activate MMP-9, and may increase permeability [55]. Other proteases, such as neutrophil elastase, may disturb the BBB Carden et al., [56] and proteolyze VE-cadherin. In this setting, generation of oxidants can inhibit endogenous anti-proteases such as α-1 anti-trypsin [57] and tissue inhibitors of metalloproteinase (TIMPs) [58], which limit junction-degrading proteases, and thus exacerbate BBB failure. The use of broad-spectrum antioxidants and MMP inhibitors (such as doxycycline and minocycline) in clinical trials [43] may preserve BBB integrity of the BBB. Several groups have described elevations of circulating inflammatory cytokines (IL-12p40, IL-17, IL-23) in patients with active MS, which decrease during remission or are reduced by IFN-β1b therapy [41]. Mechanistically, factors in sera from MS patients (in exacerbation) were found to decrease VE-cadherin and occludin expression [7], potentially contributing to the loss of BBB integrity through weaker junction organization, protein expression, and junction degradation.

Activation of p38 MAPK may influence the structural integrity of the blood brain barrier and assembly of components forming the BBB. For example, p38 MAPK activation has been shown to disturb normal assembly of occludin within tight junctions [59]. Furthermore, exposure of endothelial cells to the growth factor vascular endothelial growth factor (VEGF)-A increases permeability through phosphorylation of serine occludin (Ser490), which promotes the ubiquitination and clearance of Ser90. This loss of occludin at junctions would be expected to ‘disintegrate’ the normal junctional barrier. Interestingly, another effect of dexamethasone in ‘arterializing’ venous endothelium appears to be its effect in ‘externalizing’ cytoplasmic occludin [60], leading to a denser junction organization (Table 1). Therefore, laminar shear activation of p38 MAPK (in arterial endothelium) might enhance junction assembly, while conversely, venous shear might disassemble junctions. It is possible that orally available p38 MAPK inhibitors, (for example, SB 239063), might stabilize venous junctions and limit vascular permeability.

### MS and venous remodeling

In MS, ‘Dawson’s fingers’ are fine periventricular white matter venous lesions that appear early on in the course of MS, and are often arranged around the longitudinal axis of the central veins [8]. The venous association of this lesion has long been suspected to link venous system disturbances with the etiology of MS [61-65]. This phenomenon may represent inflammation, shear-mediated mechanical trauma, or disturbances in pressure. Anatomic reports by Schelling suggested that these lesions reflect ‘hemodynamic back jetting,’ which is theorized to be an important cause of venous injury [29]. Such lesions may be correlated with restricted outflow, which may caused by structural disturbances present in MS veins Coen et al., [66]. These structural alterations may involve switching from collagen type I to type III, which may provoke other structural abnormalities, including valve disturbances, which might alter venous hemodynamics [49]. This type of matrix remodeling might be adaptive in acute venous congestion to limit hemorrhages and iron deposition; such changes in matrix thickness or composition in ‘mature’ lesions could limit exchange or perfusion. Such non-inflammatory wall thickening is normal during aging. It is unclear whether venous structural or flow disturbances in MS might represent part of a spectrum of venous diseases seen outside of the CNS. The incidence of chronic venous disease outside of the CNS increases with age, although the age of onset for MS is between the ages of 20 and 30 years, with a female preponderance [4,67]. Like chronic venous disease, MS also shows greater prevalence in female and European populations. Interestingly, CVI, which is characterized by weak flow of venous blood, especially in the legs [68], is also characterized by collagen isoform remodeling, but shows elevation of collagen type I expression and diminished type III expression [46], increased fibrillin-1 and laminin, and overproduction of MMP1, MMP2, and MMP3 [69]. Interestingly, transforming growth factor (TGF)β1 induces endothelial apoptosis in a collagen-dependent manner, with matrix collagen type I maintaining endothelial viability despite exposure to TGF-β1 [70]. Conversely, endoglin appears to oppose TGF-β1 induced collagen synthesis by p38 MAPK activation [71], and was found to suppress TGF β1-induced collagen synthesis when ERK1/2 signaling was present. The use of p38 MAPK inhibitors, such as dilmapimod [45], might help to prevent TGF-β1-associated venous remodeling.

Both the elevation and suppression of TGF-β1 in venous structure suggest a role for TGF-β1 in CVI pathogenesis [72-75]. Active TGF-β1 increases inducible nitric oxide synthase, which dysregulates venous tone and blood flow [73]. CVI is associated with suppression of the proliferative responses of fibroblasts and smooth muscle cells to TGF-β1 [76]. TGF-β1 signaling in fibroblasts is mediated by ERK1/2 and SMAD activation [76-78]. It is unclear whether TGF modifiers, such as avotermin, might have clinical benefit in MS, as has been suggested in CVI [47]. Similarly, the angiotensin II receptor antagonist candesartan inhibits TGF-β1-induced MMP9 via Smad7 Yu et al., [79], therefore, angiotensin antagonists may also be able to suppress the vessel remodeling that can contribute to vascular abnormalities in MS.

**Figure 1:**
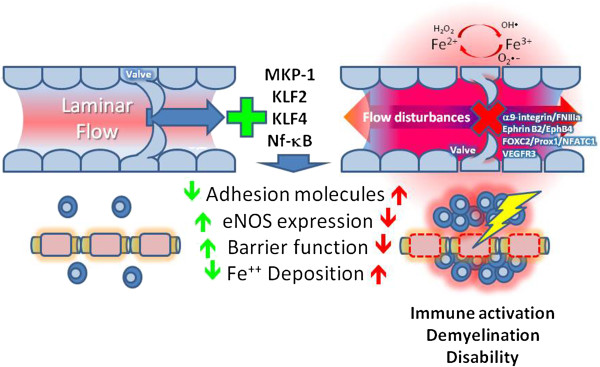
**Venous endothelial injury in neuroinflammatory disease.** Alterations in normal flow induced by changes in outflow resistance or valve failure lead to endothelial disturbances that provoke localized inflammatory responses, which may intensify immune activation, leading to demyelination and disability in MS. Flow sensors that may be dysregulated include MKP-1, KLF2, and KLF4, which control adhesion molecule, eNOS, and blood-brain barrier function and iron deposition. Venous valve structural and regulatory components that might be dysregulated in this schema include α9-integrin/fibronectin (FNIIIa), Ephrin B2/EphB4, FOXC2/Prox1/NFATC1, and VEGFR-3. Abbreviations: eNOS, endothelial nitric oxide synthase; FOXC2, Forkhead box protein C2; KLF, Krueppel-like factor; MKP, mitogen-activated protein kinase phosphatase; MS, multiple sclerosis; NFATC1, nuclear factor of activated T-cells, cytoplasmic 1; VEGFR, vascular endothelial growth factor receptor.

Bevacizumab has been shown to diminish injury in the experimental autoimmune encephalomyelitis model of MS by suppressing angiogenesis, suggesting that VEGF may play some part in the development of MS [80], Argaw *et al*. suggested that astrocytes might represent an important source of VEGF-A, which leads to the activation of eNOS and plays a significant role in the loss of BBB that occurs in MS [42]. Although not yet tested, the effects of VEGF-A on venous structure could lead to a similar loss of BBB, leading to the extravasation of lymphocytes and plasma protein, which could provoke injury and vessel remodeling. Therefore, anti-angiogenic drugs such as bevacizumab or cavtratin may find clinical applications in MS treatment (Table 1). Immunochemical and MRI methods have confirmed erythrocyte penetration in a subset of MS lesions, and the accumulation of iron-laden macrophages occurs predominantly around venules, with venous vascular lesions regularly showing iron signatures [81-85]. Iron released by extravasated erythrocytes becomes susceptible to Fenton and Haber-Weiss oxidant-generating reactions in the parenchyma, mediated by reactive oxygen species, which leads to alterations in second messenger signaling and tissue injury (Figure 1). Iron chelators (for example, desferrioxamine) (Table 1) may be effective in lowering the overall iron (and oxidant) burden.

Vessel structure in CVI is correlated with vessel stiffening; a reversed collagen expression in MS might suggest a hypercompliant venous structure. CVI is also characterized by an higher TIMP-1/MMP-2 ratio, which might favor deposition of non-elastic matrix [86]. We reported previously that MS exhibits increased expression of MMP-8 and MMP-9, which was suppressed by IFNβ1b therapy and was correlated with reduced T2-weighted lesions [41]. We also reported that addition of doxycycline, an MMP inhibitor similar to IFNβ1b, significantly reduced contrast-enhancing lesion numbers and disability scores. Lower serum MMP-9 levels correlated with fewer contrast-enhancing lesions. Furthermore, transendothelial migration of monocytes, stimulated by MS serum, was reduced in patients undergoing combination therapy with doxycycline [43]. Like MS, CVI is also characterized by increased circulating levels of MMP-9, and MMP-1, MMP-2, and TIMP-1 were also reported to be increased in CVI [Saito et al., 2001]. During chronic venous disease, the venous valves and the vessel wall exhibit monocyte and macrophage infiltration [87], which is characterized by increased expression of ICAM-1 [88]. Importantly, Takase *et al*. found inflammation of the vasa vasorum, which could provoke wall remodeling. Individuals with CVI retain more leukocytes, in which may explain the greater quantities of circulating leukocytes in CVI Bergan et al. [89]. Patients with CVI also have higher leukocyte activation and oxidant production compared with controls [90]. Powell *et al*. found more platelet-monocyte aggregates in CVI (29% versus 8%; *P* < 0.0002), while CD11b expression on monocytes in CVI was approximately twice that of controls (7.5 vs. 3.7; *P*<0.01). The presence of CVI also led to greater generation of platelet leukocyte aggregates [91]. Therefore, low or retrograde flow states, as may exist in CVI, might lead to a perilous imbalance favoring vascular inflammatory programs.

### Are there hemodynamic influences in venous vascular disturbances?

Other forms of venous restriction may also contribute to alterations in BBB. Early studies by Putnam using venous obstruction showed development of MS-like lesions [92]. More recently, Mayhan and Heistad [93] found that deliberate occlusion of the superior vena cava produced vascular solute leakage, primarily in venules. We also found recently that experimentally increased intra-abdominal hypertension (IAH) in mice (produced by abdominal volume), also caused a rapid and reversible failure of BBB (as shown by extravasation of Evans blue stain). Such changes are presumably hydrodynamic, because they resolved within 2 hours after relief of IAH [94]. Clinically, IAH above 20 mm Hg diminished venous return, and translated into increased intracranial pressure [95]. Interestingly, with respect to the potential influence of altered hemodynamics and cyclical pressure changes in the venous barrier, Shin *et al*. [96-99] showed that cyclical pressure modulates venous endothelial proliferative and barrier responses through mechanotransduction-regulated changes in fibroblast growth factor receptor/basic fibroblast growth factor and VEGF-C signaling. Interestingly, cyclical high (but not low) pressure disorganized tight (ZO-1) rather than adherens (VE-cadherin) junctional organization , and this was associated with diminished blood brain barrier.These studies provide mechanistic links between environmental pressure changes and an ‘inflammatory’ venous phenotype. It is not yet clear if such responses are unique to venous (and not arterial) endothelium.

Interestingly, Miyamoto *et al*. [100] and Yura *et al*. [101] showed that bilateral occlusion of the external jugular veins, like in mice subjected to middle cerebral artery occlusion, led to an increase in brain ischemia. Therefore, if resistance to venous outflow, either pressure-mediated or structurally mediated, provokes decreased cerebral blood flow as has been suggested [8-10], such disturbances could trigger tissue injury and demyelination (as seen in MS). An important question remains as to how downstream restriction of venous outflow might lead to a dysregulated vascular phenotype upstream of the point of insufficient venous drainage. Restriction to venous outflow would also be expected to impair normal flow-mediated vasodilatation. Impaired production of dilators such as prostacyclin, nitrous oxide, and endothelium-derived hyperpolarizing factor would lead to a retrograde volume/pressure transmission that might present as venous vascular injury. Restriction of venous outflow and congestion has been suggested to lead to distention and remodeling of venous capillaries into veins, which may have very differently structural and functional properties. Venous congestion may also provoke thrombus formation via both reduced flow and altered endothelial surface properties.

Whether intracranial venous pressure (IVP) is increased in MS remains highly controversial. McTaggart *et al*. described significant internal jugular vein (IJV) ‘flattening’ in MS and a trend toward more non-IJV collaterals [48]. Although increased intra-abdominal pressure may be produced by venous obstruction or jugular valve insufficiency, and might thenbe transmitted to the intracranial venous system, causing intracranial hypertension, [102], the significance of this mechanism in chronic cerebrospinal venous insufficiency (CCSVI) remains very controversial. Meyer-Schwickerath reported that venous pressures are normal in patients with MS [103], as measured by ophthalmodynamometry. Haacke *et al*. [2] pointed out that angioplasty in patients with MS Zamboni et al., [104] reduced venous pressure, consistent with relative pre-operative venous hypertension. Several recent reports have indicated that altered craniocervical venous outflow may also be detected in individuals diagnosed with chronic migraine [105,106], suggesting that cranial venous outflow disturbances may represent a ‘secondary’ rather than a primary phenomenon. Conversely, Lee *et al*. considered the ontogeny of several venous malformations, as they may contribute to flow disturbances in patients with MS, supporting the idea of cerebrospinal venous malformations as a primary event, which might lead to venous hypertension Lee et al., [107]. Although abnormal venous flow patterns in MS are being corrected through endovascular approaches, future studies to correlate and validate clinical outcomes and pathological mechanism are clearly needed.

### Increased intracranial venous pressure without venous leakage or demyelination: pseudotumor cerebri

Venous vascular leakage attributed to MS might be explained intuitively as the result of increased IVP, although this explanation has not been fully accepted. Of relevance to this issue is the disorder pseudotumor cerebri (PC) (also known as idiopathic intracranial hypertension), in which prolonged and demonstrably high intracranial pressures are not associated with venous leakage or demyelination. PC belongs to a set of disorders that include hydrocephalic states and spontaneous (primary) intracranial hypotension, in which the CSF circulation interfaces with the blood circulatory system. CSF moves by bulk flow and pulsatile forces (transferred from the cerebral arteries) from the ventricles into the spinal and cortical subarachnoid spaces. CSF is then largely absorbed via the arachnoid villi into the superior sagittal sinus (SSS). The pressure of the CSF (intracranial pressure, ICP) must always exceed blood pressure in the SSS for this absorption to take place. With reversal of this gradient, such as in newborns with stenosis of the jugular foramina, hydrocephalus results, as the unfused cranial sutures allow for an expansion of the ventricles, which are accumulating CSF [108]. With sutures closed, a fully myelinated, healthy brain will resist ventricular expansion, although ICP will rise, a condition predisposing to PC.

PC is a disorder mainly of females aged 15 to 45 years, with the greatest incidence in the young adult years [109]. It is characterized by high ICP, papilledema, headache, visual blurring and loss, tinnitus, retrobulbar pain, and neck stiffness [110]. Ventricular size is normal or slightly reduced. In most cases, dural venous sinus outflow obstructions or increased right atrial pressures raise IVP to the point where it challenges the ICP [111].

The MRI diagnosis of PC is partly one of exclusion of other causes of increased ICP, such as choroid plexus papilloma, cerebral edema, tumor, and obstructive hydrocephalus. Positive signs of intracranial hypertension include empty sella, bilateral increased fluid in the optic sheath, mild flattening of the posterior sclera, enhancement of the prelaminar optic nerve, distension of the periotic subarachnoid space, vertical tortuosity of the optic nerve, and gadolinium enhancement of the prelaminar optic nerve [112].

Absent from these patients are the MRI hyperintensity signals indicating demyelination. In the study of Wall *et al*., microscopic examination of brain tissue from patients with PC at autopsy showed no neuronal necrosis, gliosis, or inflammation, and no prominence of perivascular spaces or pallor of myelin in neuropil or white matter [113]. Although these findings do not exclude endothelial injury, the absence of inflammation and demyelination under conditions of prolonged venous hypertension points to the existence of factors that may protect these patients from demyelinating disease. Experimental studies focusing on high ICP states, as found in PC, would help identify these factors.

### Genes regulating venous valves

At the molecular level, if congenital or pathologic alterations in venous valve structure contribute to the etiology of CVI and other venous disturbances [114], identifying genes that control venous valve structure might provide important clues to the basis of venous pathology Bazigou *et al*. [115,116] described that venous valves are organized by interactions of several genes at different developmental stages and post-natally. The development of venous valves requires signaling from Prospero-related homeobox 1 (Prox1), vascular endothelial growth factor receptor (VEGFR)-3, and integrin α9. The binding of integrin α9 to fibronectin-IIIa is also an important structural motif necessary for venous/lymphatic valve assembly [115]. Lymphatic valve formation also involves Cnb1/NFATc1, connexin 37 and 43, and laminin-a5. Nuclear factor of activated T-cells, cytoplasmic 1 (NFATc1) also modulates cardiac valve formation [117]. The later maturation of these valves also requires Sema3A/Neuropilin-1/PlexinA1 signaling Bouvrée et al. [3]. Interestingly, Ephrin-B2 and integrin α9 were both shown to be necessary for maintenance of venous valves, as post-natal editing of these genes induced valve atresia [116]. Further, integrin α9, VEGFR3, and Prox1 were also found to be expressed in lymphatic vessel valves. Additional regulators of venous and lymphatic valve structure may include the Tie2 receptor tyrosine kinase and multiple angiopoietin ligands [116]. Tie2 signaling also appears to be responsive to altered patterns of fluid shear and is dysregulated by abnormal flow. Flow may also influence valve structure/function, as eNOS levels within valve endothelium are increased [118], and eNOS expression appears to modulate valve development, at least in aortic valves [119].

Forkhead box protein C2 (FOXC2) is another transcription factor expressed in venous and lymphatic valves, which controls their development Mellor et al., [120]. FOXC2 is dysregulated in lymphedema distichiasis [121]. Mellor *et al*. showed that individuals exhibiting mutations in FOXC2 uniformly exhibited valve disturbances in the saphenous and deep veins, contributing to venous reflux and lymphedema Mellor et al., [120]. Interestingly, although *FOXC2* gene mutations were closely associated with venous valve failure and were seen in carriers, these individuals did not have lymphedema. Currently, it is still unclear whether ‘silent’ alterations in venous/lymphatic programming genes such as *FOXC2* might contribute to venous valve failure in CNS pathology.

Among these regulators, Ephrin-B2 is usually considered to be an arterial and lymphatic specific family transmembrane ligand that binds the receptor tyrosine kinase EphB4, and participates in venous specification [122,123]. EphA2 and ephrinA1 are both persistently expressed by cultured brain endothelial cells, and treatment of brain endothelial cells with inflammatory cytokines caused the shedding of these markers into brain endothelial derived microparticles, which are small (<0.1 μm) plasma membrane vesicles [124]. Similarly, samples of control and MS serum showed increased levels of ephrin A1 and EphA2 expression in vessel structures in MS brain tissue [125]. Several vascular ephrins and Eph receptors may therefore be dysregulated in CNS inflammation, although not all of them have an influence on vascular remodeling. In the setting of CVI, IJV incompetence has been correlated with transient global amnesia [107,126-129], which may contribute to cognitive disturbances in several neurodegenerative conditions. Ephrin-B2 is strongly expressed in venous endothelium, and suppresses endothelial proliferative responses towards VEGF and Ang-2 Kim et al., [130]. By comparison, the receptors EphB2 and EphB3 are strongly expressed by arterial endothelium, and EphB/ephrin-B interactions have been suggested to modulate arteriovenous specification and separation. It is interesting to note that during inflammation, endothelial expression of EphA2 receptor and ephrin-B2 is increased [131].

### Other genes modulating venous remodeling

We have previously examined genes that were modified in cerebrovascular endothelial cells in response to serum from patients with RRMS, and found several markers that were modulated by soluble factors present in MS serum and by IFN-β1b therapy, including 14-3-3, metavinculin, myosin-3, plasminogen, reticulocalbin-2 and eticulocalbin--3, ribonuclease/angiogenin inhibitor, annexin A1, tropomyosin, and Rap1A [5]. Ferlini *et al*. (performed a gene array on chromosome 6p21.32 (human leukocyte antigen (HLA) locus) in patients exhibiting venous malformations associated with MS, and found several candidate genes that were altered including heat shock protein (HSP)A1L, HSPA1A, metabotropic glutamate receptor (GRM)4, and growth factor receptor-bound protein 2, an adaptor involved in MAPK signaling Ferlini et al., [132]. Pirmohamed et al, showed that HSPA1L might be linked with HLA-associated drug hypersensitivity [133], and increased GRM4 has also been reported in MS lesions [134]. Several genes that are associated with MS and inflammatory disease progression (VEGF, endothelin-1, IL-6, VCAM-1, ICAM-1, MMP-2, MMP-9 and PAI-1) are also modulated by alterations in mechanical stretch on the vessel wall [135]. Therefore, genes that drive venous disturbances might reflect the coincident presence of both heritable and environmental (shear/stretch) risk factors.

### Optic neuritis and vascular endothelial injury

Optic neuritis (ON) an inflammatory demyelinating disease of the optic nerve, is a common early feature of MS, and often leads to some degree of visual loss in patients. Inflammatory demyelination of the optic nerve in ON can histopathologically resemble acute MS plaques in the brain. For example, ON shows nerve sheath edema, perivenous ‘cuffing’, destruction of myelin, and vascular fluorescein leakage. Retinal VE inflammation usually precedes demyelination, and is often detected as retinal vein ‘sheathing’ [136]. Papillitis (inflammation of the optic nerve head), with increased blood flow and retinal edema, blurring of disk margins, and swollen veins can be seen in up to 30% of patients presenting with ON. A significant number of patients with ON have retrobulbar neuritis, and present with abnormal funduscopic findings.

An interesting finding in patients with MS is focal sheathing of the retinal veins (periphlebitis retinae), which includes local perivenous infiltration of lymphocytes and plasma cells [137,138], post-inflammatory peri-venular gliosis [137], and evidence of focal extravasation of plasma proteins [137]. Although the human retina has limited myelin and myelin basic proteins (limited by the lamina cribrosa [139]), or myelinating oligodendrocytes, it is still unclear as to why some patients with MS patients periphlebitis retinae. One theory to explain such findings is that other myelin-associated antigens, such as, the human natural killer-1 carbohydrate epitope and myelin-associated glycoprotein, can be expressed by retinal Müller glial cells [140,141]. However, this hypothesis cannot sufficiently explain the retinal findings in MS. To consider this issue, Engell *et al*. [142] investigated retinal venous alterations in patients with acute ON. MS was found in 41 of 76 patients examined for ophthalmologic issues; 1 patient had periphlebitis retinae and two had venous ‘sheathing’. It was concluded that altered venous structure in the retina indicated an ultimate diagnosis of MS. Therefore, because retinal venous abnormalities in patients with MS occur outside the key areas of demyelination, perivenular inflammation may represent the early event contributing to new lesions. Perivenous sheathing (periphlebitis retinae) indicates some loss of normal blood-retinal barrier. Therefore, sheathing may most be often perivenular because the venous endothelial junctions are inherently less restrictive than those of the corresponding arterial endothelium. The increased venous tendency to express adhesive inflammation-associated ECAMs in response to inflammatory or hypoxic stimuli, along with immune cell retention, may initiate or sustain exaggerated responses. In retinal endothelial monolayers (which exhibit BBB properties,) we found that the junctional solute barrier required actin microfilament assembly, was positively regulated by β-adrenoreceptor signaling [143], and was dysregulated by increased glucose levels [144]. Therefore, the BBB may be dysregulated by changes in circulating autacoids or metabolic disturbances.

### Developmental venous anomalies

Haacke *et al*. [2] suggested that that venous hypertension caused by congenital or pathologic changes could provoke the development of dural arteriovenous structural abnormalities in MS. It has even been suggested [145]that the presence of congenital venous anomalies may occur in some isolated populations (such as in Sardinia) that could contribute to more frequent or earlier-onset venous disturbances. It has been proposed that more profound vascular flow disturbances in these populations might provoke neurovascular forms of injury, which could include CCSVI or MS [145]. It is unclear whether additional risk factors are necessary to increase the penetrance of this phenotype and appearance of this condition.

### Pathophysiology of ADEM, with emphasis on venous dysfunction

ADEM is a relatively rare CNS inflammatory demyelinating disease, which affects both adults and children. ADEM typically occurs as a single-stage syndrome. It is often seen after immunization (also described as ‘post-vaccination encephalomyelitis’), and may also occur after some systemic viral infections (for example, measles). Clinically, ADEM produces a variety of symptoms, including fever, headache, meningismus, seizures, loss of sensation/tingling, visual loss, weakness or paralysis, loss of coordination, involuntary spasms, and loss of sphincter control. Neuropathologically, ADEM exhibits scattered focal demyelination, which is usually limited to the perivenous areas. The underlying neuropathological defects in ADEM can affect both the brain and spinal cord, with MRI often revealing large and diffuse or multifocal lesions. This appearance differs from that of MS in that MS lesions are focal, smaller, and confluent [146]. The MRI lesions of ADEM involve both gray and white matter [147].

Neuropathological studies in ADEM have shown merged regions of perivenular demyelination throughout the cerebral hemispheres, brainstem, cerebellum, and spinal cord. Although these lesions are usually most numerous in the white matter, they can affect deeper layers of the cerebral cortex, thalamus, hypothalamus, and other gray-matter areas within the brain. Microscopically, ADEM affects small distended veins enclosed within parenchymal infiltrates of reactive microglia, lymphocytes, macrophages, and occasionally neutrophils, associated with demyelination [39].

Although the details of ADEM pathogenesis remain only partially understood, interactions between inflamed and activated underlying cerebral venous endothelium and activated leukocytes play major roles in its development. Following activation of the immune system, either because of molecular mimicry or sensitization against the self-antigens following a viral infection, myelin basic protein-reactive lymphocytes can interact with the venous endothelium [148]. Such interactions between the inflamed venous endothelium and the activated leukocytes can disrupt the normal functional and anatomical integrity of the cerebral venous endothelium, and eventually promote the transendothelial migration of leukocytes and release of neuroinflammatory mediators such as cytokines and chemokines. Further research into the immunopathogenesis of ADEM versus MS reveals that T helper (Th)1-related and Th2-related chemokines are generated during both ADEM and MS. ADEM shows upregulation of chemokines for neutrophils (CXCL1, CXCL7), monocytes/T cells (CCL3, CCL5), Th1 cells (CXCL10), and Th2 cells (CCL1, CCL22, and CCL17) [39]. Further, the involvement of MMP-9 [149] and increased serum levels of soluble ICAM-1 in the pathogenesis of ADEM has been shown [150], which places more emphasis on endothelial disturbances underlying ADEM pathology. Interestingly, the inflammatory demyelinating lesions of ADEM do not form near arterial vessels. This finding itself lends support to the concept that inherent venous (rather than arterial) endothelial anatomic or functional abnormalities drives ADEM.

## Conclusions

The roles of anatomical and functional abnormalities of the cerebral venous endothelium in the pathogenesis of human CNS inflammatory diseases such as MS and ADEM often remain unrecognized, underinvestigated, and untreated. Rather than these diseases simply being the result of structural disturbances of veins, together with the combined hemodynamic (low/abnormal flow, pressure/congestion), programmatic (arterial, venous, valvular) and environmental (metabolic, hypoxic) stresses to which venous endothelial cells are exposed may render them particularly susceptible to inflammatory activation, contributing to several neurovascular pathologies. Presently, markers of arterial and venous endothelial specification and the role of each cell type in inflammation are now receiving more attention. A more thorough understanding of such mechanisms based on the developmental, cellular, and molecular mechanisms underlying the hemodynamic disturbances of these conditions will open many new therapeutic targets for debilitating diseases such as Alzheimer’s disease and MS.

## Abbreviations

ADEM: Acute disseminated encephalomyelitis; BBB: Blood-brain barrier; CCSVI: Chronic cerebrospinal venous insufficiency; CNS: Central nervous system; CSF: Cerebrospinal fluid; CVI: Chronic venous insufficiency; ECAM: Endothelial cell adhesion molecule; eNOS: Endothelial nitric oxide synthase; FOXC2: Forkhead box protein C2; GRM: Metabotropic glutamate receptor; HDAC: Histone deacetylase; HLA: Human leukocyte antigen; HSP: Heat shock protein; IAH: Intra-abdominal hypertension; ICAM: Intercellular adhesion molecule; ICP: Intracranial pressure; IFN: Interferon; IJV: nternal jugular vein; IL: Interleukin; IVP: ntracranial venous pressure; KLF: Krueppel-like factor; LPS: Lipopolysaccharide; MAPK: Mitogen-activated protein kinase; MKP: mitogen-activated protein kinase phosphatase; MMP: Matrix metalloproteinase; MRI: Magnetic resonance imaging; MS: Multiple sclerosis; NAWM: Normal-appearing white matter; NMDA: N-methyl-D-aspartate; ON: Optic neuritis; PAI: Plasminogen activator inhibitor; PC: Pseudotumor cerebri; PPMS: Primary progressive multiple sclerosis; Prox1: Prospero-related homeobox 1; RRMS: Relapsing-remitting multiple sclerosis; SSS: Superior sagittal sinus; Th: T helper; TIMP: Tissue inhibitor of metalloproteinase; TM: Thrombomodulin; TNF: Tumor necrosis factor; VCAM: Vascular cell adhesion molecule; VE: Vascular endothelial; VEGF: Vascular endothelial growth factor.

## Competing interests

The authors declare no competing interests.

## Authors’ information

JJSA and CVG are members of the Molecular and Cellular Physiology Department, LSUHSC-Shreveport; LP is a member of the Pathology Department, LSUHSC-Shreveport, IT is a member of the Department of Microbiology and Immunology, LSUHSC-Shreveport, and AM is a member of Department of Neurology, LSUHSC-Shreveport.
